# Analysis of US Teen Driving After Using Marijuana, 2017

**DOI:** 10.1001/jamanetworkopen.2020.30473

**Published:** 2020-12-23

**Authors:** Li Li, Guoqing Hu, David C. Schwebel, Motao Zhu

**Affiliations:** 1 Division of Epidemiology, College of Public Health, The Ohio State University, Columbus; 2 Department of Epidemiology and Health Statistics, Hunan Provincial Key Laboratory of Clinical Epidemiology, Xiangya School of Public Health, Central South University, Changsha, Hunan, China; 3 Department of Psychology, University of Alabama at Birmingham; 4 Center for Injury Research and Policy, The Abigail Wexner Research Institute at Nationwide Children’s Hospital, Columbus, Ohio; 5 Department of Pediatrics, College of Medicine, The Ohio State University, Columbus

## Abstract

This cross-sectional study examines the prevalence of and factors associated with driving after using marijuana among US adolescents.

## Introduction

Marijuana use impairs cognitive abilities necessary for safe driving, including reaction time, road lane–tracking ability, and attention maintenance. In 2018, 45.3% of US residents aged 12 years or older reported having used marijuana in their lifetime; 10.1% were recent users.^1^ Although US high school seniors reporting driving after using marijuana (DAUM) decreased slightly from 14.6% in 2001 to 12.4% in 2011,^2^ given recent trends in the legalization of marijuana for recreational and medicinal use, teenagers may show decreases in perceived harmfulness of marijuana use and increases in general marijuana use.^3,4^ This study aims to estimate the prevalence of and factors associated with DAUM among US teenaged drivers.

## Methods

Cross-sectional survey data were retrieved from the 2017 national Youth Risk Behavior Survey. The Nationwide Children’s Hospital institutional review board deemed this study exempt from review and waived informed patient consent because the data did not include personal identifiers. This study followed the Strengthening the Reporting of Observational Studies in Epidemiology (STROBE) reporting guideline.

The outcome was self-reported DAUM at least once in the month before survey completion. Unlike previous research that estimated DAUM prevalence among all drivers and ignored whether drivers had a history of marijuana use, this study considered 2 denominators: all teen drivers and drivers currently using marijuana, defined as marijuana use at least once in the past month. Therefore, results are comparable to previous research but also specifically consider behavior of current marijuana users. Crude and adjusted prevalence ratios (PRs) and 95% CIs for DAUM were estimated using Poisson regression with robust variance estimation. Covariate selection was based on literature review and backward selection method (*P* > .20 removed). Data were weighted to adjust for nonresponse, high school grade, sex, race/ethnicity, and survey design. Statistical significance was set at α = .05 and all tests were 2-tailed. Statistical analysis was performed using Stata version 14.0 (StataCorp) from February to October 2020.

## Results

Of the 6816 students aged 14 years or older who responded in 2017 and indicated driving in the past month, 3399 (weighted percentage, 50%) were male, 3027 were White (weighted percentage, 56%), and 969 (weighted percentage, 12.7% [95% CI, 11.3%-14.1%]) reported DAUM in the past month, which was more than twice the rate of reported drinking and driving (327 drivers; weighted percentage, 5%). Among the 1590 teen drivers who reported current marijuana use, 795 (weighted percentage, 48.8% [95% CI, 44.7%-53.0%]) reported DAUM. Male youths and older participants had higher DAUM weighted percentages than their counterparts (male: 52.9% [95% CI, 47.7%-58.2%]; female: 44.5% [95% CI, 39.5%-49.4%]) (aged ≥18 years: 63.3% [95% CI, 56.1%-70.5%]; aged 17 years: 57.1% [95% CI, 52.2%-62.0%]; aged 16 years: 39.5% [95% CI, 31.3%-47.8%]; aged 15 years: 28.7% [95% CI, 22.7%-34.7%]; aged 14 years: 42.8% [95% CI, 25.7%-59.8%]). Compared with other races, Hispanic students had the highest DAUM prevalence with 271 drivers (weighted percentage, 14.5% [95% CI, 11.9%-17.0%]) among general teen drivers, but White students had the highest rate with 339 drivers (weighted percentages, 53.0% [95% CI, 46.9%-59.2%]) among drivers currently using marijuana (Table).

**Table.** DAUM During the Past Month Among US High School Students, 2017 Youth Risk Behavior Survey^a^

Variables	All teen drivers^b^		Drivers currently using marijuana^c^	
	Unweighted No. (weighted %)	DAUM, % (95% CI)^d^	Unweighted No. (weighted %)	DAUM, % (95% CI)^d^
Overall	6816	12.7 (11.3-14.1)	1590	48.8 (44.7-53.0)
Age, y				
14	364 (6)	7.7 (4.1-11.2)	57 (4)	42.8 (25.7-59.8)
15	1237 (19)	6.8 (4.9-8.7)	206 (14)	28.7 (22.7-34.7)
16	1871 (28)	11.0 (8.9-13.0)	429 (29)	39.5 (31.3-47.8)
17	2101 (29)	15.3 (13.3-17.3)	538 (31)	57.1 (52.2-62.0)
≥18	1243 (18)	19.1 (15.3-22.8)	360 (22)	63.3 (56.1-70.5)
Sex				
Female	3401 (50)	11.1 (9.4-12.9)	777 (49)	44.5 (39.5-49.4)
Male	3399 (50)	14.2 (12.6-15.7)	805 (51)	52.9 (47.7-58.2)
Missing	16	NA	8	NA
Race/ethnicity				
White	3027 (56)	11.7 (9.9-13.6)	635 (50)	53.0 (46.9-59.2)
Black	1181 (12)	12.8 (10.3-15.3)	312 (14)	39.9 (32.7-47.2)
Hispanic	1835 (23)	14.5 (11.9-17.0)	494 (28)	44.8 (40.8-48.7)
Other^e^	685 (9)	13.3 (9.1-17.5)	134 (8)	51.9 (39.8-64.1)
Missing	88	NA	15	NA
Current alcohol use^f^				
No	3790 (64)	3.9 (3.1-4.8)	336 (25)	38.7 (31.7-45.7)
Yes	2042 (36)	26.5 (23.9-29.1)	963 (75)	52.6 (48.0-57.3)
Missing	984		291	
Binge drinking^g^				
No	5245 (83)	6.4 (5.3-7.4)	766 (55)	39.2 (33.7-44.7)
Yes	984 (17)	35.7 (31.8-39.7)	561 (45)	59.8 (53.7-65.9)
Missing	587	NA	263	NA
Current cigarette use^f^				
No	5955 (90)	8.3 (7.0-9.6)	1083 (69)	41.9 (36.6-47.2)
Yes	653 (10)	45.4 (40.2-50.6)	430 (31)	63.3 (58.9-67.6)
Missing	208	NA	77	NA
Riding with a drinking driver^f^				
No	5578 (83)	9.0 (7.8-10.2)	1100 (69)	42.4 (37.0-47.9)
Yes	1233 (17)	30.0 (26.1-33.9)	489 (31)	63.2 (57.7-68.7)
Missing	5	NA	1	NA
Seatbelt use				
Never, rarely, or sometimes	1088 (14)	26.8 (22.9-30.6)	420 (24)	56.0 (51.0-60.9)
Always or most of the time	5633 (86)	10.4 (8.9-11.9)	1144 (76)	46.9 (42.0-51.8)
Missing	95	NA	26	NA
Drinking and driving^h^				
No	5997 (94)	8.6 (7.4-9.8)	1192 (83)	40.4 (35.6-45.2)
Yes	327 (5)	60.1 (53.2-67.1)	213 (15)	84.5 (77.9-91.1)
Missing	404	NA	141	NA
Marijuana early use				
No	NA	NA	1219 (77)	44.8 (39.8-49.8)
First use <13 y	NA	NA	357 (23)	62.4 (56.7-68.0)
Missing	NA	NA	14	NA

Abbreviations: DAUM, driving after using marijuana; NA, not applicable.

^a^ Three states were excluded from analysis because missing values on driving after using marijuana were more than 50%: Maryland (96%), Pennsylvania (56%), and Utah (100%).

^b^ Participants who indicated driving in the past month and aged 14 years or older.

^c^ Participants aged 14 years or older who indicated driving in the past month and using marijuana at least once in the past month.

^d^ Prevalence of participants reporting DAUM at least once in the past month.

^e^ Includes American Indian/Alaska Native, Asian, Native Hawaiian or other Pacific Islander, and Multiple Non-Hispanic/Latino.

^f^ Engagement in this behavior reported at least once in the past month.

^g^ Defined as reporting having 4 or more drinks of alcohol in a row (female youths) or 5 or more drinks of alcohol in a row (male youths) at least once in the past month.

^h^ Teens who reported driving after using alcohol at least once in the past month. The percentages do not sum to 100% because conflicting responses were omitted, such as when respondents indicated they did not drive in the past month but also reported they drove after using alcohol.

Multiple regression analyses suggested that alcohol and cigarette use were associated with DAUM among all teen drivers (prevalence ratio [PR] for current alcohol use, 2.94 [95% CI, 2.06-4.22]; PR for current cigarette use, 2.23 [95% CI, 1.69-2.95]) but not among those who currently use marijuana (PR for current alcohol use, 0.92 [95% CI, 0.70-1.20]; PR for current cigarette use, 1.14 [95% CI, 0.97-1.36]). Binge drinking and drinking and driving were associated with higher prevalence of DAUM among all teen drivers (PR for current binge drinking, 1.71 [95% CI, 1.32-2.20]; PR for current drinking and driving, 1.71 [95% CI, 1.42-2.06]) and those who currently use marijuana (PR for current binge drinking, 1.23 [95% CI, 0.99-1.50]; PR for current drinking and driving, 1.54 [95% CI, 1.29-1.84]) (Figure).

**Figure.**
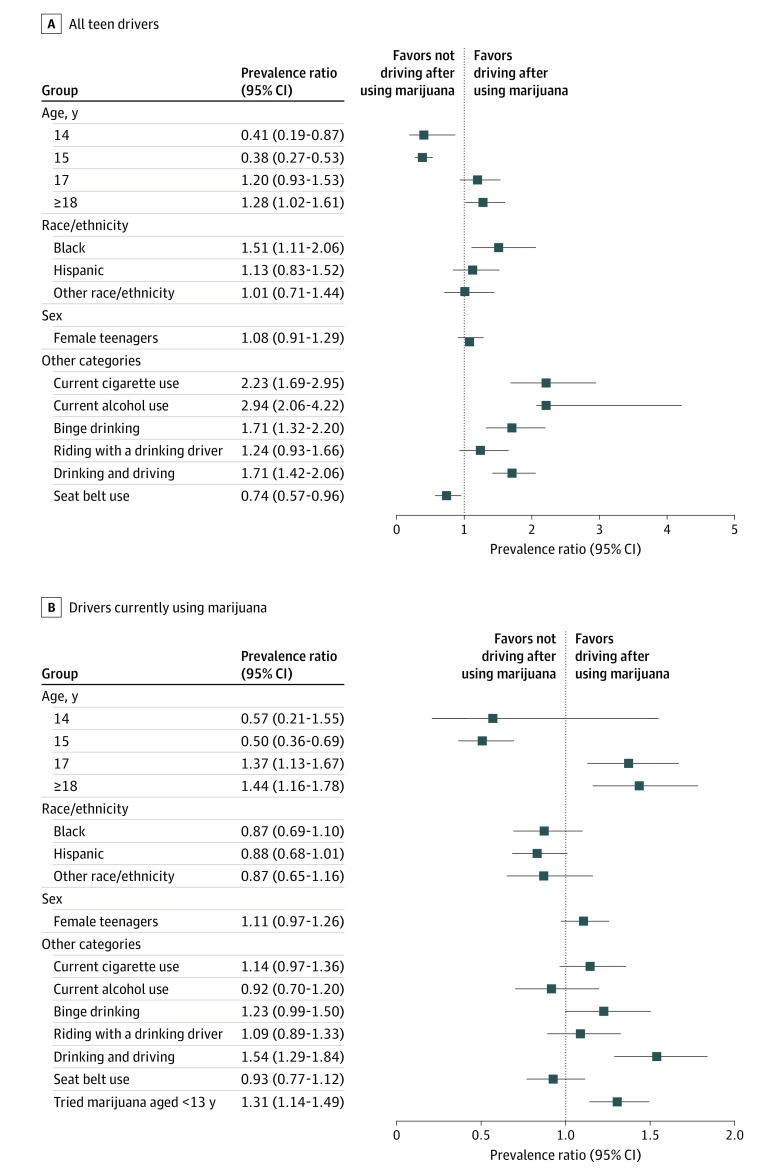
Prevalence Ratios of Driving After Using Marijuana in the Past Month, 2017 Youth Risk Behavior Survey

## Discussion

Our study found that almost half (48.8%) of teen drivers who currently use marijuana reported DAUM, which is 17% higher than the rate found in a study of first-year college students in 2012.^5^ We also found that the prevalence of DAUM (12.7%) was more than double the prevalence of drinking and driving (5.0%), perhaps reflecting teens’ perception that DAUM is less dangerous and more acceptable than driving after using alcohol. Policies such as zero tolerance of THC (tetrahydrocannabinol) while driving and increased age limits for legal marijuana consumption might help alter social norms among teens.

Although we found a higher prevalence of DAUM among male youths than female youths, the differences were not statistically significant. This contrasts with previous research among high school seniors in 2009 to 2012^2^ and first-year college students with current marijuana use in 2012.^5^ Sex disparities in teen marijuana use are decreasing,^6^ and intervention programs should target reduced DAUM among both sexes.

This study had some limitations. We were limited by asking only about DAUM frequency in the past month, and we could not assess a driver’s degree of impairment.

As US states legalize medical and recreational marijuana use, teens may misperceive the risk of marijuana use^4^ and DAUM. More than 1 in 8 teen drivers reported DAUM in the past month. Almost half of drivers who currently use marijuana engaged in DAUM. Strategies to adopt and enforce policies that change social norms and increase perceived harmfulness offer promise to mitigate the risks associated with DAUM.
